# CD32 Ligation Promotes the Activation of CD4^+^ T Cells

**DOI:** 10.3389/fimmu.2018.02814

**Published:** 2018-11-30

**Authors:** María Pía Holgado, Inés Sananez, Silvina Raiden, Jorge R. Geffner, Lourdes Arruvito

**Affiliations:** ^1^Instituto de Investigaciones Biomédicas en Retrovirus y SIDA, Universidad de Buenos Aires, CONICET, Buenos Aires, Argentina; ^2^Unidad I, Departamento de Clínica Médica, Hospital de Niños Pedro de Elizalde, Buenos Aires, Argentina; ^3^Departamento de Microbiología, Parasitología e Inmunología, Facultad de Medicina, Universidad de Buenos Aires, Buenos Aires, Argentina

**Keywords:** T cells, FcγR, IgG, cytokines, proliferation, activation

## Abstract

Low affinity receptors for the Fc portion of IgG (FcγRs) represent a critical link between innate and adaptive immunity. Immune complexes (ICs) are the natural ligands for low affinity FcγRs, and high levels of ICs are usually detected in both, chronic viral infections and autoimmune diseases. The expression and function of FcγRs in myeloid cells, NK cells and B cells have been well characterized. By contrast, there are controversial reports about the expression and function of FcγRs in T cells. Here, we demonstrated that ~2% of resting CD4+ T cells express cell surface FcγRII (CD32). Analysis of CD32 expression in permeabilized cells revealed an increased proportion of CD4+CD32+ T cells (~9%), indicating that CD4+ T cells store a CD32 cytoplasmic pool. Activation of CD4+ T cells markedly increased the expression of CD32 either at the cell surface or intracellularly. Analysis of CD32 mRNA transcripts in activated CD4+ T cells revealed the presence of both, the stimulatory FcγRIIa (CD32a) and the inhibitory FcγRIIb (CD32b) isoforms of CD32, being the CD32a:CD32b mRNA ratio ~5:1. Consistent with this finding, we found not only that CD4+ T cells bind aggregated IgG, used as an IC model, but also that CD32 ligation by specific mAb induced a strong calcium transient in CD4+ T cells. Moreover, we found that pretreatment of CD4+ T cells with immobilized IgG as well as cross-linking of CD32 by specific antibodies increased both, the proliferative response of CD4+ T cells and the release of a wide pattern of cytokines (IL-2, IL-5, IL-10, IL-17, IFN-γ, and TNF-α) triggered by either PHA or anti-CD3 mAb. Collectively, our results indicate that ligation of CD32 promotes the activation of CD4+ T cells. These findings suggest that ICs might contribute to the perpetuation of chronic inflammatory responses by virtue of its ability to directly interact with CD4+ T cells through CD32a, promoting the activation of T cells into different inflammatory profiles.

## Introduction

Receptors for the Fc portion of IgG (FcγRs) are widely express in immune cells and mediate a large array of effector and immunomodulatory mechanisms that influence both innate and adaptive responses (1). FcγRs are classified into two main types that include different members. Type I FcRs belong to the immunoglobulin receptor superfamily and are represented by the canonical Fcγ receptors, including FcγRI, FcγRII, and FcγRIII. Type II FcRs belong to the family of C-type lectin receptors, and include CD209 (DC-SIGN) and CD23 (2). Based on the signaling motifs expressed in the cytoplasmic domains, type I FcγRs are classified as stimulatory or inhibitory receptors, which are associated with immunoreceptor tyrosine activation motifs (ITAM) or immunoreceptor tyrosine inhibition motifs (ITIM), respectively (1, 3, 4). Stimulatory type I receptors include FcγRI (CD64), FcγRIIa (CD32a), FcγRIIc (CD32c), and FcγRIIIa (CD16), while inhibitory type I receptors only include FcγRIIb (CD32b) (5). Activating FcγRs signal through an ITAM motif that is either present in their intracytoplasmic domain or in associated signaling subunits, such as the FcRγ chain. These ITAM-containing structures allow FcγRs, once aggregated by multimeric ligands, to stimulate signaling cascades via SRC and SYK kinases promoting cell activation. The inhibitory receptor FcγRIIb possesses instead an ITIM motif in its intracytoplasmic domain, which allows this receptor to recruit the SHIP1 phosphatase that counteracts the signaling cascades initiated by activating FcγRs. Therefore, the co-expression of these divergent receptors, which share almost identical ligand-binding domains, establishes a threshold of cell activation (3, 6–8).

The expression pattern and the function of type I FcγR have been well characterized in innate immune cells and B cells (2, 8, 9). By contrast, the expression and function of CD32 in CD4+ T cells remain controversial. The most cited reviews in the field assume that CD4+ T cells do not express FcγRs (1, 10, 11), however, contrasting observations have been published. It has been reported that resting CD4+ T cells do not express CD32 (12–14), while other studies have shown that a minor fraction of resting CD4+ T cells (1–5%) actually expresses CD32 (15, 16). It has also been reported that activation of CD4+ T cells promotes an increased expression of CD32 (17, 18). Moreover, early studies have claimed that more than 80% of resting CD4+ T cells expresses high amounts of CD32 as an intracellular pool (18, 19, 20). Interestingly, recent observations suggested that CD32 is a marker of a CD4+ T cell population that contains an HIV reservoir harboring replication-competent proviruses (14), however this finding remains controversial (16, 21).

Immune complexes (ICs) are the natural ligands of low affinity FcγRs (1). High levels of ICs are found in chronic viral infections and autoimmune diseases (22). Moreover, a large body of evidence suggest that ICs play a key role not only in the induction of tissue injury, but also in the promotion of T cell responses (23–28). The mechanisms underlying the ability of ICs to promote the stimulation of T cells have not been clearly defined yet. However, it is usually assumed that they are related to the ability of ICs to interact with FcγRs expressed by macrophages and dendritic cells improving antigen presentation to both CD4+ and CD8+ T cells (27, 28). Interestingly, the enhanced antigen immunogenicity conferred by ICs has been adopted as strategy to improve the therapeutic efficiency of vaccines in experimental models of viral infectious diseases (27, 29, 30).

Here, we showed that a minor fraction of resting CD4+ T cells express cell surface CD32. Activation of CD4+ T cells promoted a marked increase in the expression of CD32 either at the cell surface or intracellularly. Analysis by qRT-PCR on activated CD4+ T cells revealed the presence of mRNA transcripts for both, the stimulatory FcγRIIa (CD32a) and the inhibitory FcγRIIb (CD32b) isoforms, being the CD32a:CD32b mRNA ratio ~5:1. Consistent with this finding, we found that cross-linking of CD32 by immobilized IgG or specific mAb directed to CD32 enhanced both, the proliferative response and a wide pattern of cytokines by CD4+ T cells stimulated by PHA or anti-CD3 mAb. Overall, our observations suggest that ICs might perpetuate the chronic inflammatory response in patients with autoimmunity and/or chronic viral infections, not only by stimulating innate immune cells, but also by directly interacting with CD4+ T cells via CD32a.

## Materials and methods

### Subjects

Buffy coat was obtained from 1 unit of blood collected from 45 donors (average 34 years, range 25–42 years), and processed immediately after volunteer's donations. None of the donors had any hereditary disorders, hematologic abnormalities, or infectious diseases. The local ethics committee has approved this study and informed consent was obtained from all donors.

### Peripheral blood mononuclear cell (PBMCs) isolation

PBMCs were obtained from buffy coats by Ficoll-Hypaque gradient centrifugation (GE Healthcare Life Sciences).

### Monocytes and CD4+ T cell isolation

Monocytes and/or CD4+ T cells were enriched from buffy coats by using the RosetteSep human monocyte and/or CD4+ T cells enrichment cocktails (Stem Cell Technologies) respectively, following the manufacturer's protocols. The purity determined by flow cytometry was always >97%.

### Cell sorting

Both CD32+CD4+ and CD32-CD4+ T cell subsets were purified by cell sorting with a FACSAria Fusion flow cytometer (BD Biosciences). Briefly, previously activated PBMCs were stained with anti-CD3 PerCP, anti-CD4 APC, and CD32 PE-Cy7 monoclonal antibodies (mAbs, all from Biolegend) and sorted yielding the following subpopulations CD4+CD32+ T cells and CD4+CD32- T cells. Cells were collected into RPMI 1640 medium containing 50% FBS and washed twice prior to further studies. The purity determined by flow cytometry was always >99% for each subset. Cells were resuspended in TRIzol reagent (Thermo Fisher) and used for qRT-PCR.

### Flow cytometry

Freshly isolated or *in vitro*-cultured cells were stained with anti-CD3, CD4, CD8, CD14, CD19, CD25, HLA-DR, PD-1, and Tim-3 mAb (all from Biolegend). Staining of CD32 was performed by using two different anti-human CD32 mAb: FUN.2 clone (mouse anti-human CD32 PE or PECy7 conjugated, Biolegend) and IV.3 clone (mouse anti-human CD32 purified antibody, Stem Cell Technologies) that was revealed with a secondary PE goat Fab2 anti-mouse IgG (DAKO). Intracellular detection of Ki-67 antigen with anti-Ki-67 antibody was performed using fixed and permeabilized cells following the manufacturer's instructions (BD Biosciences). Control samples were incubated with an isotype-matched antibody. For the determination of CD32 expression, PE and/or PE-Cy7 mouse IgG2b kappa (Biolegend) were included as isotype controls, using a threshold value ≤ 0.2 in all cases. When CD32 was determined by using unconjugated IV.3 clone, a purified mouse IgG2b kappa (Biolegend) was used as isotype control followed by a secondary PE anti-mouse antibody. Dead cells were excluded by forward and side scatter characteristics. Statistical analyses were based on at least 100,000 events gated on the population of interest. The data were acquired using a FACSCanto II (Becton Dickinson) and analyzed with FlowJo software.

### Fluorescence microscopy

Briefly, purified cells were incubated with monoclonal anti-CD32 Alexa Fluor 647 and anti-CD4 Alexa Fluor 488 or anti-CD14 Alexa Fluor 488 as indicated (all from Biolegend). Then, cells were washed twice and allowed to adhere on polylysine-coated coverslips. After which, cells were fixed with 4% paraformaldehyde in Phosphate Buffered Saline (PBS) for 12 m at 4°C, washed twice and treated with 10 mM glycine for 10 m at room temperature. The coverslips mounted with DAPI Fluoromount-G (SouthernBiotech) were studied in a Nikon Eclipse Ti-S L100 fluorescence microscope using a Plan Apochromat 100 × 1.40 NA oil immersion objective.

### PBMCs culture

PBMCs (1 × 10^6^/ml) were stimulated with IL-2 (20 ng/ml, Peprotech), coated anti-CD3 (aCD3, 10 μg/ml, Beckman Coulter) plus soluble anti-CD28 (aCD28, 1 mg/ml, BD Biosciences), or unstimulated, and cultured for 36 h in medium RPMI 1640 (Gibco) supplemented with 10% heat-inactivated fetal bovine serum (FBS, Natocor), 2 mM L-glutamine (Gibco), 100 U/ml penicillin (Gibco), and 0.1 mg/ml streptomycin (Gibco). In some experiments, cells were treated with phytohemagglutinin (PHA, 4 μg/ml; Sigma-Aldrich) and cultured for 36 h. After that, cells were washed twice and analyzed by flow cytometry.

### Real-time quantitative RT-PCR

Total RNA was extracted using TRIzol reagent following manufacturer's instructions. Subsequently, RNA was treated with RQ1 RNAse-free DNAse (Promega) and reverse transcripted using M-MLV Reverse Transcriptase (Sigma-Aldrich). PCR analysis for both, CD32a (FcγRIIa) and CD32b (FcγRIIb) isoforms was performed with a real-time PCR detection system (StepONE-Plus Applied Biosystems) using 5 × HOT FIREPol® EvaGreen® qPCR Mix Plus (ROX) (Solis BioDyne Corp) as a fluorescent DNA-binding dye. GAPDH was used as housekeeping gene. The amplification protocol was as follows: 1 cicle at 95°C for 10 m and 40 cycles of denaturation at 95°C for 15 s, annealing at 56°C for 15 s and extension at 72°C for 1 m. The melting curve was also performed. Each sample was evaluated by triplicate.

The following primer sets were used: FcγRIIa forward primer, 5′-ATCATTGTGGCTGTGGTCATTGC-3′ and reverse primer, 5′- TCAGGTAGATGTTTTTATCATCG-3′; and FcγRIIb forward primer, 5′- GGGATCATTGTGGCTGTG-3′ and FcγRIIb reverse primer, 5′-ATTAGTGGGATTGGCTG-3′. GAPDH forward primer, 5′-GAGTCAACGGATTTGGTCGT-3′ and reverse primer 5′-TTGATTTTGGAGGGATCTCG-3′. Primer sets yielded a single product of the correct size.

As a source of cDNA for standard curves to which all samples were normalized (calibrator), monocytes were isolated as previously described. Standard curves and relative quantification was performed as previously published (31). In short, the threshold cycle (C_T_) values, determined by the StepONE Plus software v2.3, were used to calculate and plot a linear regression curve to evaluate the quality of the standard curve. The slope of this line was used to determine the efficiency of the reaction *(E)*. From the C_T_'s and the efficiencies obtained, the normalized value was calculated with the following formula: E_T_^CpT(C)−CpT(S)^:E_R_^CpR(C)−CpR(S)^, in which E_T_ is the efficiency of the PCR of the target gene (FcγRIIa or FcγRIIb2); E_R_, the efficiency of the PCR of the reference gene (GAPDH); CpT(C), the measured C_T_ of the target gene determined for standard or calibrator (FcγRIIa or FcγRIIb of one selected monocyte sample for all measurements); CpT(S), the measured C_T_ of the target gene determined for the sample (donor of interest); CpR(C), the measured C_T_ of the reference gene of the calibrator or standard; and CpR(S), the measured C_T_ of the reference gene of the sample.

### Ligation of CD32

CD32 was cross-linked in purified CD4+ T cells by using two different approaches:

1. Ligation with coated IgG (cIgG): We immobilized IgG on plastic plates, as described (32). Human IgG (500 μg/ml, Sigma-Aldrich) was added to each well of a 48 multiwell plate (GBO). After overnight incubation, plates were washed 3 times with PBS to yield immobilized IgG. Purified CD4+ T cells (1 × 10^6^/ml) were seeded on cIgG overnight.
2. Ligation with specific anti-CD32 mAb: Purified CD4+ T cells (1 × 10^6^/ml) were preincubated for 30 m with anti-CD32 mAb (30 μg/ml, clone IV.3; Stem Cell Technologies) and then stimulated by the addition of F(ab′)2 fragment goat anti mouse IgG (Fab'2, 50 μg/ml; Jackson ImmunoResearch) for additional 30 min. Afterwards, cells were seeded on uncoated plates overnight.

After CD32 ligation by one of the two aforementioned approaches, cells were stimulated with suboptimal doses of PHA (0.5 μg/ml) or anti-CD3 coated beads (0.025 μg/ml, Miltenyi Biotec) and cultured during 5 d. As controls, CD4+ T cells were added to uncoated plates and stimulated with PHA or anti-CD3 coated beads, according to the experiment. The proliferative response and the cytokine levels were analyzed by flow cytometry and ELISA, respectively.

### Neutralization assay

To block the CD32 receptor expressed by CD4+ T cells, cells were incubated with a blocking anti-CD32 mAb (30 μg/ml, clone IV.3) before being exposed to cIgG (IV.3 plus cIgG). An isotype-matched antibody was used as a control.

### Binding of heat-aggregated IgG (aIgG)

IgG aggregates (aIgG) were prepared by heating human IgG (25 mg/ml) for 12 m at 63°C. Then, aIgG was centrifuged at 10,000 g for 5 m and the precipitate was discarded. Resting or activated purified CD4+ T cells were incubated with different doses of aIgG (50, 100, or 400 μg/ml, as indicated), or serum free-medium for 2 h at 37°C. Then, cells were washed three times and stained with biotinylated anti-human IgG Fc (Biolegend) for 30 m at 4°C followed by streptavidin PerCP and anti-CD4 V500. Binding of aIgG was analyzed by flow cytometry. In blocking experiments, cells were pretreated with the blocking antibody IV.3 clone (30 μg/ml) for 30 m at 4°C, before the addition of aIgG (IV.3 plus aIgG).

### Calcium mobilization assay

CD32-triggered calcium transients were analyzed by flow cytometry on purified CD4+ T cells using Fluo-3,AM probe (Molecular Probes, Thermo Fisher). Briefly, 1 × 10^6^ resting CD4+ T cells were stained with anti-CD4 mAb APC. After washing twice, cells were treated with 5 μM Fluo-3,AM for 20 m at 37°C. Then, the sample was immediately loaded onto the flow cytometer for calcium baseline measurement during 30 s. Afterward, the purified anti-CD32 mAb (IV.3 clone, 30 μg/ml) was added and calcium measurement was performed for other 30 s. Subsequently, the cells were incubated at 37°C for 15 m. After this time, Fab′2 (50 μg/ml) was added and calcium mobilization was measured immediately and incubated at 37°C for another 60 s. Finally, the fluorescence was recorded during an additional period of 60 s. As a positive control, we treated cells with ionomycin (Sigma-Aldrich). A time-based gate was used for the analysis in gated CD4+ T cells.

### STAT5 and STAT6 phosphorylation

Purified CD4+ T cells (1 × 10^6^) were cultured in the presence of cIgG (500 μg/ml) or IV.3 (30 μg/ml) plus cIgG for 18 h. Then, cells were re-stimulated with PHA (0.5 μg/ml) and cultured for 3 d. After this period, cells were fixed and permeabilized (BD Biosciences) according to the manufacturer's protocols. Cells were washed twice with PBS and stained with anti-CD3 FITC and anti-STAT 5 (pY694) Alexa Fluor 647 or anti-STAT6 (pTyr641) Alexa Fluor 647, for 30 m at room temperature. Cells were then analyzed on a BD FACS Canto II flow cytometer.

### ELISA

Levels of IL-2, IL-5, IL-10, IFN-γ, TNF-α (BD Biosciences), and IL-17 (Biolegend) were quantified in cells supernatants by ELISA following manufacturer's recommendations.

### Statistical analysis

Statistical analysis was performed using GraphPad Prism 6 software. Two groups were compared using the Wilcoxon signed-rank test or Mann–Whitney *t*-test as appropriated. Three or more groups were compared using the Kruskall–Wallis test followed by Dunn's multiple comparison tests. A *p* < 0.05 was considered statistically significant.

## Results

### Resting CD4+ T cells express CD32

**Figure 1:**
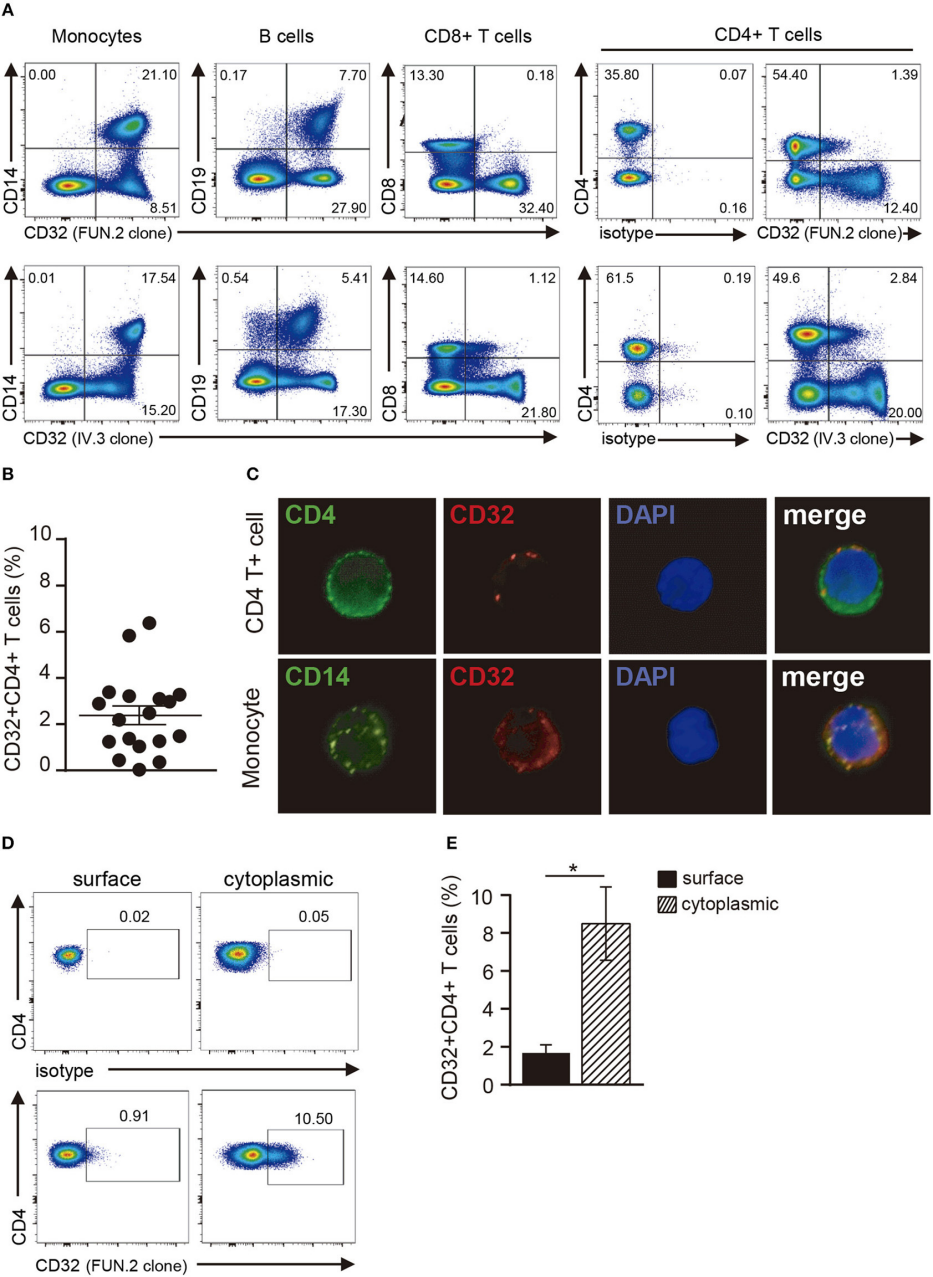
Analysis of CD32 expression in resting CD4+ T cells. **(A)** Representative dot plot of CD32 cell surface expression in monocytes (CD14+), B cells (CD19+), CD8+ and CD4+ T cells from a healthy adult donor using two different anti-CD32 mAb (FUN.2 and IV.3 clones) analyzed by flow cytometry. Surface isotype control labeling was set to stringent criteria. Results are expressed as percentages on PBMCs. **(B)** Frequency of CD32+ cells on gated CD4+ T cells from healthy adults using the FUN.2 clone mAb by flow cytometry. **(C)** Fluorescence microscopy of CD32 expression in purified CD4+ T cells and monocytes (green: CD4 or CD14, red: CD32). Nuclear counterstain with DAPI was used. Representative images are shown at x300. **(D)** Representative dot plot of cell surface and cytoplasmic CD32 expression in permeabilized resting CD4+ T cells. Surface and cytoplasmic isotype controls are shown. **(E)** Frequency of cell surface and cytoplasmic CD32 expression on resting CD4+ T cells. Results are expressed as percentages on CD4+ T cells. Representative experiments are shown in **(A,C,D)**. Mean ± SEM of n donors are shown in **(B)** (*n* = 18) and **(E)** (*n* = 9). *p < 0.05. Wilcoxon matched-pairs signed rank test was used for analysis in **(E)**.

In a first set of experiments, we explored the expression of CD32 in resting CD4+ T cells by using two different anti-CD32 mAbs (FUN.2 and IV.3 clones). CD32 expression was also analyzed on monocytes, B cells, and CD8+ T cells. As described (33–35), monocytes and B cells showed a high expression of CD32, by contrast only a minor fraction of CD8+ T cells and CD4+ T cells expressed CD32. In fact, we found that ~2.4% ± 0.4 of CD4+ T cells were shown to be positive for the expression of CD32 (*n* = 18; Figures 1A–C). We then analyzed the cytoplasmic expression of CD32 in CD4+ T cells. Results in Figures 1D,E show that ~8.5% ± 1.9 of permeabilized cells expressed CD32 (*n* = 9), indicating that CD4+ T cells store an intracellular pool of this receptor.

### Increased expression of CD32 in activated CD4+ T cells

**Figure 2:**
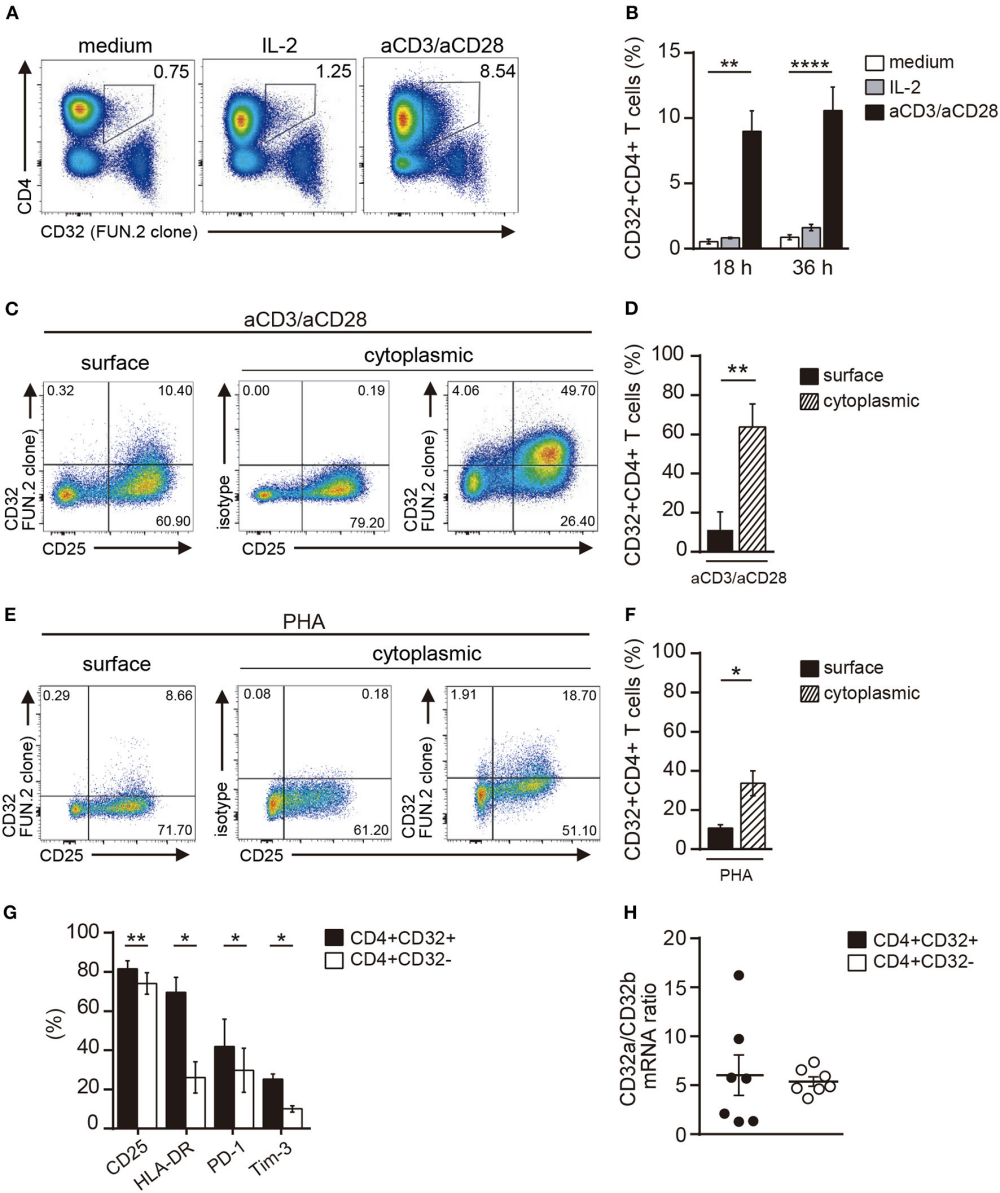
Activation of CD4+ T cells results in an increased expression of CD32. **(A,B)** PBMCs were cultured with medium (controls), IL-2 (20 ng/ml) or immobilized anti-CD3 (10 μg/ml) plus soluble anti-CD28 (1 mg/ml) (aCD3/aCD28) antibodies, during 18 or 36 h. The frequency of CD32+CD4+ T cells was analyzed by flow cytometry. **(A)** Representative dot plots of cell surface expression of CD32 in CD4+ T cells analyzed at 36 h of culture. Results are expressed as percentages on total lymphocytes. **(B)** Frequency of CD32+ cells on gated CD4+ T cells at 18 and 36 h of culture. **(C–F)** PBMCs were activated with either aCD3/aCD28 antibodies **(C,D)** or PHA (4 μg/ml, **E,F**) for 36 h. Then, cell surface or intracellular expression of CD32 was analyzed. Cell surface expression of CD25 was also assessed. Cytoplasmic isotype control is shown in **(C,E)**. **(G)** Cell frequency of the different markers analyzed in CD32+CD4+ and CD32-CD4+ T cells. Results are expressed as percentage on each CD4+ T cell subset analyzed. **(H)** CD32a/CD32b mRNA ratio in activated CD32-CD4+ and CD32+CD4+ T cell subsets analyzed by qRT-PCR. Representative experiments are shown in **(A,C,E)**. Mean ± SEM of *n* donors are shown in **(B)** (*n* = 7), **(D)** (*n* = 7), **(F)** (*n* = 7), **(G)** (*n* = 8), and **(H)** (*n* = 7). ^*^p < 0.05, ^**^p < 0.01, ^****^p < 0.0001. Kruskal–Wallis test followed by Dunn's multiple comparison was used for analysis in **(B)**. Wilcoxon matched-pairs signed rank test was used for analysis in **(D,F,G,H)**.

Next, we examined whether T cell activation was able to modulate CD32 expression. PBMCs were stimulated with IL-2 or with antibodies directed to CD3 and CD28 for 18 or 36 h. Then, the expression of CD32 was analyzed. Treatment with aCD3/aCD28 antibodies markedly increased cell surface expression of CD32 at either 18 or 36 h of culture while IL-2 induced no increase of CD32 expression (Figures 2A,B). We also observed that activation of CD4+ T cells by aCD3/aCD28 antibodies resulted in an increased pool of cytoplasmic CD32 (Figures 2C,D).

**Figure 3:**
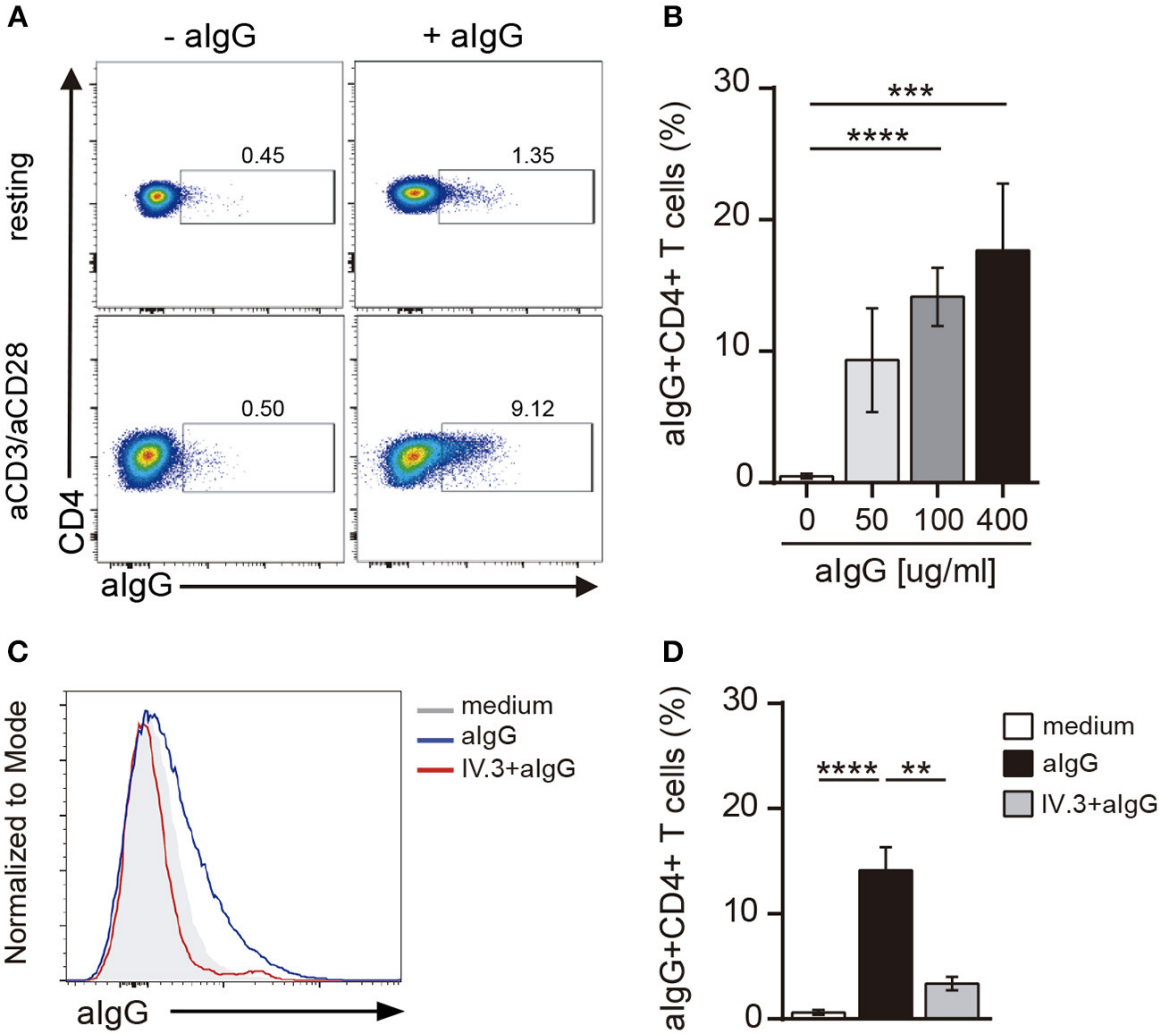
CD4+ T cells bind aIgG. **(A)** Resting or activated purified CD4+ T cells were incubated with aIgG (400 μg/ml) or serum-free medium for 2 h. Then, cells were washed and the binding of aIgG was analyzed by flow cytometry. **(B)** Activated CD4+ T cells were incubated with increasing concentrations of aIgG (50, 100, and 400 μg/ml) or serum-free medium. Data show the percentage of aIgG+CD4+ T cells. **(C,D)** Activated CD4+ T cells were treated with or without an anti-CD32 blocking antibody (IV.3 clone, 30 μg/ml) during 30 m. After washing, cells were incubated with 100 μg/ml of aIgG or serum-free medium. **(C)** Representative histograms of aIgG percentage are shown. **(D)** Percentage of aIgG+CD4+ T cells is shown. Representative experiments are shown in **(A,C)**. Mean ± SEM of *n* donors are shown in **(B)** (*n* = 8) and **(D)** (*n* = 8). ^**^p < 0.01, ^***^p < 0.001, ^****^p < 0.0001. Kruskal–Wallis test followed by Dunn's multiple comparison was used for analysis in **(B)**. Mann–Whitney test was used for analysis in **(D)**.

Activation of CD4+ T cells by PHA also promoted an increased expression of CD32 (Figures 2E,F). Because the expression of CD32 was increased during CD4+ T cell activation, we analyzed whether CD32 expression was associated with the induction of T cell activation markers. Results in Figure 2G show that the subpopulation of CD32+ CD4+ T cells was enriched not only in the expression of activation markers such as CD25 and HLA-DR, but also in the expression of the inhibitory receptors PD-1 and Tim-3, which are usually associated with an exhausted CD4+ T cell phenotype (36). Further experiments were performed to evaluate the relative expression of CD32 isoforms (CD32a and CD32b) by qRT-PCR in sorted CD32+CD4+ and CD32-CD4+ T cell subsets, after activation with aCD3/aCD28 antibodies. We found that the mRNA levels for CD32a were significantly higher compared with CD32b, being the CD32a:CD32b mRNA ratio ~5:1 in both cell subsets (*n* = 7, Figure 2H). On the other hand, and consistent with the higher expression of CD32 observed in activated cells, we found that cell activation also resulted in an increased ability of CD4+ T cells to bind aIgG, in a dose-dependent mode (Figures 3A,B). As expected, binding of aIgG was prevented by the anti-CD32 blocking IV.3 mAb (14.1% ± 2.2 vs. 3.4% ± 0.6, for aIgG and IV.3 plus aIgG, respectively, *p* < 0.01, *n* = 8; Figures 3C,D).

### Ligation of CD32 promotes the activation of CD4+ T cells

**Figure 4:**
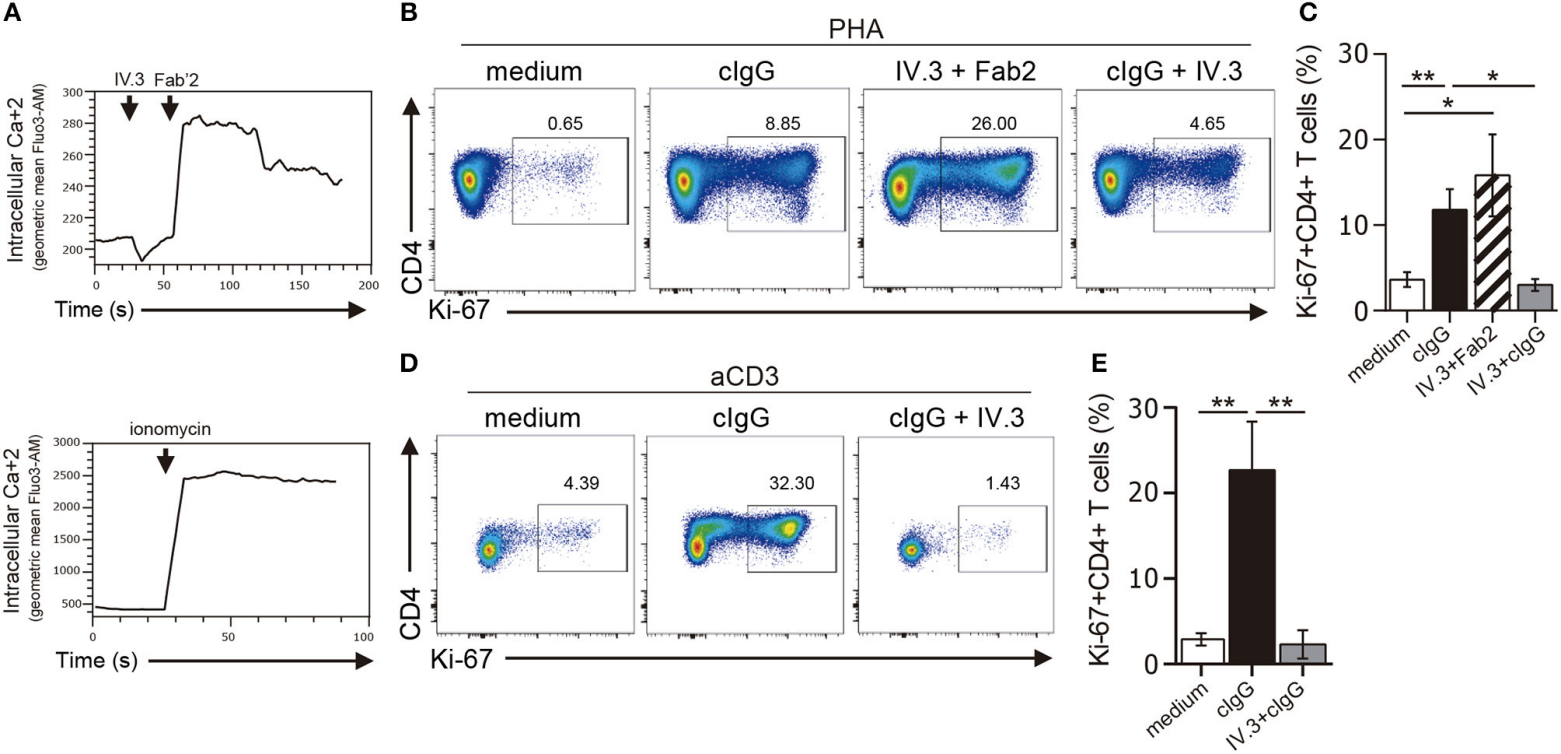
Stimulation through CD32 promotes the expansion of CD4+ T cells activated by suboptimal doses of different stimuli. **(A)** Purified CD4+ T cells labeled with fluo-3,AM fluorescent probe were treated with an anti-CD32 mAb (IV.3 clone) for 15 m. Then a F(ab′)2 fragment goat anti mouse IgG was added to induce the cross-linking of bound IV.3 mAb. Induction of cytosolic calcium transients was analyzed by flow cytometry at three different times: before and after addition of IV.3 and after stimulation with Fab′2 (upper panel). Cells were treated with ionomycin as a positive control (lower panel). A representative experiment is shown (*n* = 5). Geometric mean of fluo-3,AM vs. time is depicted. **(B–E)** Purified CD4+ T cells (1 × 10^6^) were cultured in the presence of medium (control), cIgG or IV.3 plus a Fab′2 antibody directed to mouse IgG, for 18 h. When IV.3 was used as blocking mAb, cells were pretreated with IV.3 clone, before being exposed to cIgG. Then, cells were stimulated with a suboptimal concentration of PHA (0.5 μg/ml, **B,C**) or anti-CD3 coated beads (0.025 μg/ml, **D,E**) and cultured for 5 d. Proliferative response was evaluated by using Ki-67 staining. **(B–D)** Representative dot plots of CD4 and Ki-67 staining are shown. Results are expressed as cell percentages on gated CD4+ T cells. **(C–E)** Percentage of Ki-67+CD4+ T cells analyzed by flow cytometry. Representative experiments are shown in **(B,D)**. Mean ± SEM of n donors are shown in **(C)** (*n* = 10) and **(E)** (*n* = 6). ^*^p < 0.05, ^**^p < 0.01. Kruskal–Wallis test followed by Dunn's multiple comparison was used for analysis in **(C,E)**.

Ligation of CD32 has shown to induce calcium signaling in myeloid cells and platelets (37, 38). To analyze the functionality of CD32, we first analyzed its ability to induce calcium transient in CD4+ T cells. In these experiments, cells were preincubated with the IV.3 mAb and then treated with a Fab'2 polyclonal goat anti-mouse IgG (39). Results in Figure 4A (upper panel) show that CD32 ligation caused the induction of a strong calcium transient.

We then analyzed whether CD32 ligation was able to promote the proliferation of CD4+ T cells. In these experiments, isolated CD4+ T cells were stimulated, or not, with cIgG or IV.3 plus a Fab′2 polyclonal goat anti-mouse IgG (IV.3 plus Fab′2) for 18 h. Then, cells were re-stimulated with a suboptimal dose of PHA (0.5 μg/ml) and cultured during 5 d. The proliferative response was analyzed by studying the expression of the Ki-67 antigen as a proliferation marker. We found that ligation of CD32 does not induce proliferation of CD4+ T cells (data not shown), but significantly increased the proliferative response induced by PHA. As expected, pretreatment of CD4+ T cells with the blocking IV.3 mAb significantly prevented the enhancing effect induced by cIgG on the proliferation of CD4+ T cells (Figures 4B,C). No proliferation was observed in cells treated only with IV.3 or a secondary goat Fab2 anti-mouse IgG, compared with those cells cultured in medium alone (data not shown). Consistent with these findings, we found that CD32 ligation also stimulated the proliferative response of CD4+ T cells triggered by beads coated with anti-CD3 mAb (28.2% ± 7.1 and 3.5% ± 0.9, for CD4+ T cells pretreated, or not, with cIgG, respectively, *p* < 0.01; *n* = 6). Moreover, this enhancing effect was significantly inhibited in presence of the mAb IV.3 (Figures 4D,E).

**Figure 5:**
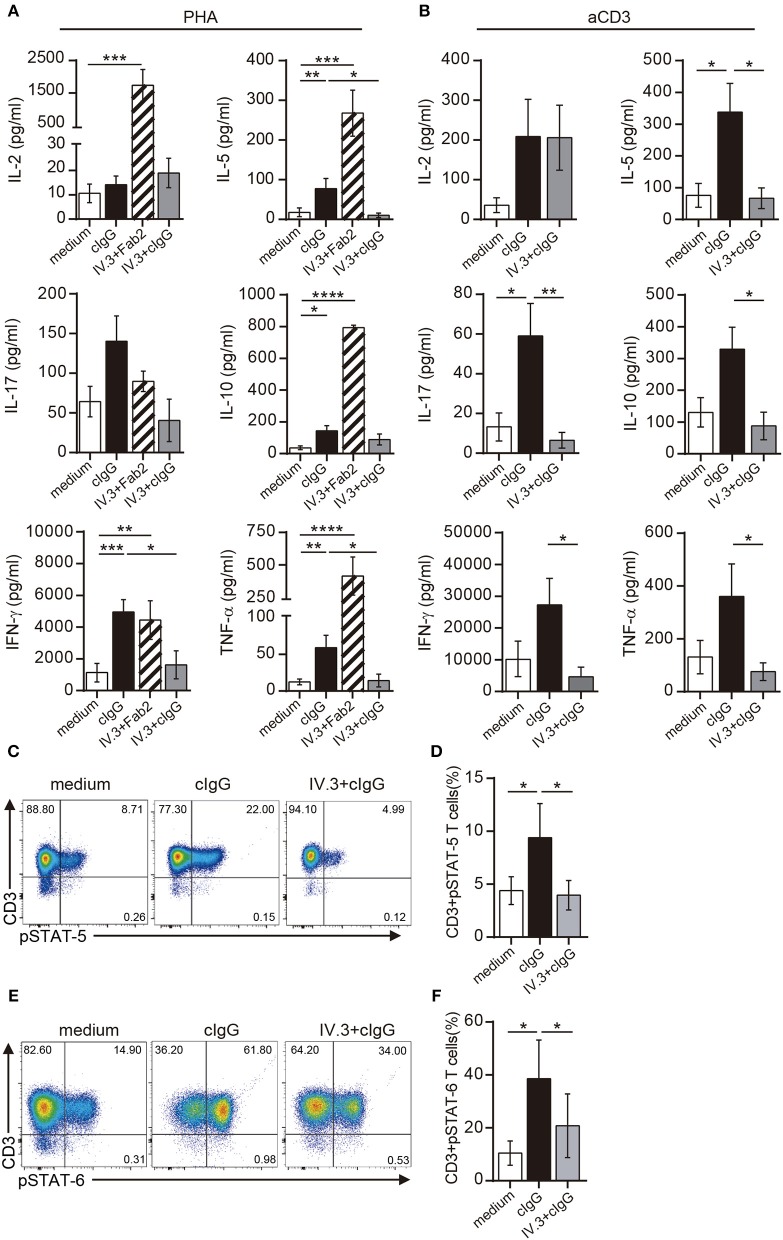
Stimulation through CD32 promotes the release of cytokines in CD4+ T cells activated by suboptimal doses of different stimuli. **(A,B)** Purified CD4+ T cells (1 × 10^6^) were cultured in the presence of medium (control, white bar), cIgG (black bar) or IV.3 plus a Fab′2 antibody directed to mouse IgG (striped bar), for 18 h. When IV.3 was used as blocking mAb, cells were pretreated with IV.3 clone, before being exposed to cIgG (gray bar). Then, cells were stimulated with a suboptimal concentration of PHA (0.5 μg/ml, **A**) or anti-CD3 (0.025 μg/ml, **B**) and cultured for 5 d. Levels of cytokines were quantified in the culture supernatant by ELISA. **(C–F)** Purified CD4+ T cells (1 × 10^6^) were cultured in the presence of medium or cIgG for 18 h. When IV.3 was used as blocking mAb, cells were pretreated with IV.3 clone, before being exposed to cIgG. Then, cells were stimulated with a suboptimal concentration of PHA (0.5 μg/ml) and cultured for 3 d. STAT5 **(C,D)** and STAT6 **(E,F)** phosphorylation was analyzed by flow cytometry. Results are expressed as percentage on CD4+ T cells. Representative experiments are shown in **(C,E)**. The mean ± SEM of n experiments is shown in **(A)** (*n* = 10), **(B)** (*n* = 6), **(D)** (*n* = 5), and **(F)** (*n* = 5). ^*^p < 0.05, ^**^p < 0.01, ^***^p < 0.001, ^****^p < 0.0001. Mann–Whitney test was used for analysis in **(A,B)**. Kruskal–Wallis test followed by Dunn's multiple comparison was used for analysis in **(D,F)**.

Not only the proliferation, but also the production of a wide pattern of cytokines was stimulated by CD32 ligation in CD4+ T cells cultured with suboptimal doses of PHA. However, the two different approaches for CD32 ligation displayed a different stimulating ability (Figure 5A). Cross-linking of IV.3 with a Fab′2 goat anti-mouse IgG (IV.3 plus Fab′2) resulted in a strong enhancement in the production of IL-2 (1738.0 pg/ml ± 490.6 vs. 10.6 pg/ml ± 3.8, *p* < 0.001), IL-5 (267.9 pg/ml ± 57.9 vs. 17.9 pg/ml ± 11.0, *p* < 0.001), IL-10 (793.1 pg/ml ± 15.8 vs. 36.7 pg/ml ± 11.8, *p* < 0.0001), IFN-γ (4443.0 pg/ml ± 1218 vs. 1132.0 pg/ml ± 591.1, *p* < 0.05), and TNF-α (414.6 pg/ml ± 147.0 vs. 12.3 pg/ml ± 4.0, *p* < 0.001), compared with control cells (*n* = 10). There was also a non-significant increase in the production of IL-17. No cytokine secretion was detected in cells treated only with IV.3 or a secondary goat Fab2 anti-mouse IgG (data not shown). On the other hand, exposure of CD4+ T cells to cIgG did not increase IL-2 levels, but significantly enhanced the secretion of IL-5 (77.9 pg/ml ± 25.3; *p* < 0.01), IL-10 (142.9 pg/ml ± 33.6; *p* < 0.05), IFN-γ (4954.0 pg/ml ± 772.8; *p* < 0.001), and TNF-α (59.1 pg/ml ± 16.9; *p* < 0.01), compared with control cells. Pretreatment of CD4+ T cells with the IV.3 mAb significantly inhibited the stimulatory effect of cIgG on the production of IL5, IFN-γ, and TNF-α (Figure 5A).

Similar findings were observed in CD4+ T cells upon stimulation with anti-CD3 coated beads. Exposure of CD4+ T cells to cIgG significantly increased the secretion of IL-5 (338.1 pg/ml ± 90 vs. 75.7 pg/ml ± 37.4; *p* < 0.01), IL-17 (59.1 pg/ml ± 16.4 vs. 13.2 pg/ml ± 7.0; *p* < 0.01), compared with control cells. It also promoted the secretion of IL-2 (208.5 pg/ml ± 93.8 vs. 35.8 pg/ml ± 18.6), IL-10 (329.2 pg/ml ± 69.4 vs. 130.4 pg/ml ± 46.2), IFN-γ (27,323 pg/ml ± 8285 vs. 10,126 pg/ml ± 5704), and TNF-α (360.2 pg/ml ± 123.3 vs. 130.8 pg/ml ± 36.2) compared with control cells, even this enhancing effect did not reach statistical significance (*n* = 6). As expected, pretreatment of CD4+ T cells with the IV.3 mAb significantly inhibited the stimulatory effect of cIgG on the production of IL5, IL-10, IL-17, IFN-γ, and TNF-α (Figure 5B).

IL-2 supports the proliferation of CD4+ T cells being the activation of STAT5 one of the earliest events in IL-2 signaling through the high affinity IL-2 receptor (40). Results in Figures 5C,D show that cIgG promoted STAT5 phosphorylation in CD4+ T cells cultured with suboptimal concentrations of PHA (9.4% ± 3.2 vs. 4.4% ± 1.3, for cIgG treated vs. untreated cells, *p* < 0.05). Moreover, we found that cIgG also enhanced the phosphorylation of STAT6, which is the main transcription factor responsible for the development of Th2 cells (41) (38.6% ± 14.5 vs. 10.5% ± 4.6, for cIgG treated vs. untreated cells, *p* < 0.05). As expected, pretreatment with the blocking IV.3 mAb significantly prevented the stimulatory effect induced by cIgG on the phosphorylation of both, STAT5 and STAT6 (Figure 5E,F, *n* = 5).

## Discussion

The expression and function of FcγRs in innate immune cells and B cells have been clearly defined (2, 9, 10). Although it is generally assumed that T cells do not express FcγRs (8, 12, 42) a more detailed analysis of early published studies shows contradictory findings (17–19). In this study, we found not only that a minor fraction of resting CD4+ T cells expresses CD32, but also that T cell activation induces a marked up-regulation of CD32 expression in either the cell surface or the cytoplasmic compartment. The role of CD32, if any, in determining the function of CD4+ T cells remains undefined. Here we reported that, contrasting with the inhibitory effect of CD32 in B cell responses (3, 43), it promotes the activation of CD4+ T cells.

Our observations indicating that a small percentage of resting CD4+ T cells expresses cell surface CD32 are in agreement with those data recently reported by Martin et al. (16) in studies directed to analyze the role of CD32+CD4+ T cells as viral reservoirs in HIV-infected patients. Moreover, in accordance with Sandilands et al. (19), we also found that resting CD4+ T cells express a cytoplasmic pool of CD32. However, while these authors reported that more than 90% of resting CD4+ T cells stores an intracellular pool of CD32, we found that only ~9% of resting CD4+ T cells contain a cytoplasmic pool of CD32. The reasons underlying this discrepancy are unclear. On the other hand, and in agreement with early works (17, 18), we detected that activation of CD4+ T cells increases cell surface expression of CD32, reaching a peak at 36 h of culture. We also found that CD32 expressing CD4+ T cells displayed an activated phenotype characterized by the expression of activation markers such as CD25 and HLA-DR as well as by the expression of the inhibitory receptors PD-1 and Tim-3, which are usually associated with an exhausted CD4+ T cell phenotype. Accordingly, with these findings, we observed that activated CD4+ T cells bind higher amounts of aIgG compared with resting cell. Our results showing that concentrations of aIgG as high as 100 μg/ml were unable to saturate CD32 binding capacity are consistent with the fact that CD32 is a low- affinity FcγR.

It has long been known that IgG ICs suppress humoral immunity. This effect is mediated through the interaction of the Fc fragment of IgG antibodies with the only FcγR isoform expressed by B cells, FcγRIIb. In fact, cross-linking of FcγRIIb with the B cell receptor has shown to increase the threshold for B cell activation, inhibiting antibody production (3, 43, 44). Interestingly, our findings revealed for the first time that cross-linking of CD32 efficiently promoted the activation of CD4+ T cells stimulated by either suboptimal doses of PHA or anti-CD3 mAb. Our results showing that ligation of CD32 promotes the activation of CD4+ T cells suggest that CD32a is the predominant type of FcγRII expressed in this cell population. The similarity of the extracellular domains of CD32b and CD32a does not enable the differentiation of these receptors by flow cytometry because there are no commercially available antibodies that can distinguish between them. The present study provides evidence indicating the presence of both CD32 mRNA isoforms in activated CD4+ T cells, being CD32a the predominant one. In fact, we found that activation of CD4+ T cells resulted in the expression of both CD32a and CD32b transcripts in a ratio of 5:1, respectively.

Ligation of CD32 induced by seeding CD4+ T cells on immobilized IgG or by treatment with anti-CD32 mAbs promoted neither cell proliferation nor cytokine production by CD4+ T cells. However, both stimuli significantly enhanced the proliferative response and the production of a wide array of cytokines by CD4+ T cells treated with suboptimal doses of PHA or anti-CD3 mAb. We found a higher production of cytokines associated with the development of Th1 (IL-2, IFN-γ, and TNF-α), Th2 (IL-5), and Th17 (IL-17) profiles, suggesting that CD32 ligation does not promote a particular signature in CD4+ T cells. Because ICs are the most important ligands for FcγRs, our observations might be relevant in those disorders associated with the presence of high levels of circulating ICs, such as chronic infections (45–47), autoimmune diseases (48–51) and cancer (52–54). Our results suggest that ICs might promote the development of inflammatory responses, by acting directly on CD4+ T cells via CD32a. Moreover, our results could contribute to better explain why antigens contained in ICs promote a stronger T cell responses compared with non-complexed antigens, a phenomenon usually attributed to the ability of ICs to stimulate antigen presentation through major histocompatibility complex class II and class I molecules (27–30). Further studies are required to confirm both, the ability of ICs to activate CD32 in presence of the high concentrations of monomeric IgG found in plasma and the *in vivo* relevance of our observations.

It should be note that the ability of ICs to modulate the function of CD4+ T cells could be related not only to the expression of CD32, but also to the expression of CD16 (FcγRIII). Early studies showed the expression of CD16 in a small number of peripheral T cells in healthy individuals (12, 55). Moreover, more recent studies performed by Chauhan and coworkers (13, 56–58) reported that CD4+ T cell activation leads to the up-regulation of CD16 expression. In a first paper, the authors showed that ligation of CD16 in CD4+ T cells by ICs induces a co-stimulatory signal promoting IFN-γ production (56). In a second study, they reported that ICs isolated from systemic lupus erythematosus patients interact with CD16 expressed by CD4+ T cells and induce Syk phosphorylation, providing a co-stimulatory signal to T cells in the absence of CD28 signal. This mechanism was shown to be able to promote the development of Th1 and Th17 cells (57). Finally, the authors reported that stimulation of CD4+ T cells by ICs induced, not only the up-regulation of endosomal toll-like receptors (TLRs), but also the accumulation of TLR9 on the cell surface (13).

Overall, our results suggest that CD32 ligation promoted CD4+ T cell activation. Whether CD16 might acts in concert with CD32 to induce the activation of CD4+ T cells remains to be addressed. Because autoimmune diseases are usually associated with the formation and tissue deposition of immune complexes (42), we hypothesize that they might contribute to tissue damage, not only by activating inflammatory mechanisms mediated by innate immune cells, but also by stimulating the chronic activation of CD4+ T cells. Finally, the fact that CD32 ligation can influence CD4+ T cell activation may have another important clinical implication. Modulating the ability of a therapeutic IgG antibody to bind to activating or inhibitory CD32 could promote the balance in favor of CD4+ T cell activation or suppression. Cellular activation is a desired characteristic in immunotherapies directed to cancer or in vaccine generation against infectious diseases. However, when it comes to chronic inflammation or autoimmune diseases, the contrary effect is sought, being the suppression of the immune response a requirement for the induction of immune tolerance. Thus, therapeutic strategies based on monoclonal IgG antibodies must take into account the potential modulation on CD4+ T cell function through their Fc portion as a novel key player.

## Ethics statement

This study was approved by the Ethics Committee of the University of Buenos Aires, Buenos Aires, Argentina, in accordance with the Declaration of Helsinki (Fortaleza, 2013). Written informed consent was obtained from all donors.

## Author contributions

MH designed and performed the experiments, analyzed data, and wrote the manuscript. IS performed some experiments and analyzed data. SR analyzed data. JG contributed to experimental design, data analysis, and revised the manuscript. LA designed the experiments, analyzed data, and wrote the manuscript.

### Conflict of interest statement

The authors declare that the research was conducted in the absence of any commercial or financial relationships that could be construed as a potential conflict of interest.

## Abbreviations

aCD3/aCD28: anti-CD3 and anti-CD28 stimulation
aIgG: aggregated IgG
ART: antiretroviral treatment
cIgG: coated IgG
Fab'2: F(ab')2 fragment goat anti mouse IgG
FBS: Fetal Bovine Serum
FcγRs: Fc γ receptors
ICs: Immune Complexes
ITAM: immunoreceptor tyrosine activation motifs
ITIM: immunoreceptor tyrosine inhibition motifs
mAb: monoclonal antibody
PBMCs: Peripheral Blood Mononuclear Cells
PBS: Phosphate Buffered Saline
PHA: phytohemagglutinin
STAT: Signal transducer and activator of transcription
TCR: T cell receptor.

## References

[1] Bournazos S Chow SK Abboud N Casadevall A Ravetch JV. Human IgG Fc domain engineering enhances antitoxin neutralizing antibody activity. J Clin Invest. (2014) 124:725–9. 10.1172/JCI72676 24401277 PMC3904629

[2] Pincetic A Bournazos S DiLillo DJ Maamary J Wang TT Dahan R. Type I and type II Fc receptors regulate innate and adaptive immunity. Nat Immunol. (2014) 15:707–16. 10.1038/ni.2939 25045879 PMC7430760

[3] Amigorena S Bonnerot C Drake JR Choquet D Hunziker W Guillet JG. Cytoplasmic domain heterogeneity and functions of IgG Fc receptors in B lymphocytes. Science (1992) 256:1808–12. 1535455 10.1126/science.1535455

[4] Swanson JA Hoppe AD. The coordination of signaling during Fc receptor-mediated phagocytosis. J Leukoc Biol. (2004) 76:1093–103. 10.1189/jlb.0804439 15466916

[5] Muta T Kurosaki T Misulovin Z Sanchez M Nussenzweig MC Ravetch JV. A 13-amino-acid motif in the cytoplasmic domain of Fc gamma RIIB modulates B-cell receptor signalling. Nature (1994) 369:340. 10.1038/369340a0 8183374

[6] Ono M Bolland S Tempst P Ravetch JV. Role of the inositol phosphatase SHIP in negative regulation of the immune system by the receptor Fc(gamma)RIIB. Nature (1996) 383:263–6. 10.1038/383263a0 8805703

[7] Ravetch JV Lanier LL. Immune inhibitory receptors. Science (2000) 290:84–9. 10.1126/science.290.5489.84 11021804

[8] Nimmerjahn F Ravetch JV. Fcgamma receptors as regulators of immune responses. Nat Rev Immunol. (2008) 8:34–47. 10.1038/nri2206 18064051

[9] Guilliams M Bruhns P Saeys Y Hammad H Lambrecht BN. The function of Fcgamma receptors in dendritic cells and macrophages. Nat Rev Immunol. (2014) 14:94–108. 10.1038/nri3582 24445665

[10] Ravetch JV Bolland S. IgG Fc receptors. Annu Rev Immunol. (2001) 19:275–90. 10.1146/annurev.immunol.19.1.275 11244038

[11] Hogarth PM. Fc receptors are major mediators of antibody based inflammation in autoimmunity. Curr Opin Immunol. (2002) 14:798–802. 10.1016/s0952-7915(02)00409-0 12413532

[12] Sandor M Lynch RG. Lymphocyte Fc receptors: the special case of T cells. Immunol Today (1993) 14:227–31. 8517922 10.1016/0167-5699(93)90168-K

[13] Chauhan AK. Human CD4(+) T-cells: a role for low-affinity fc receptors. Front Immunol. (2016) 7:215. 10.3389/fimmu.2016.00215 27313579 PMC4887501

[14] Descours B Petitjean G Lopez-Zaragoza JL Bruel T Raffel R Psomas C. CD32a is a marker of a CD4 T-cell HIV reservoir harbouring replication-competent proviruses. Nature (2017) 543:564–7. 10.1038/nature21710 28297712

[15] Mantzioris BX Berger MF Sewell W Zola H. Expression of the Fc receptor for IgG (Fc gamma RII/CDw32) by human circulating T and B lymphocytes. J Immunol. (1993) 150:5175–84. 8496609

[16] Martin GE Pace M Thornhill JP Phetsouphanh C Meyerowitz J Gossez M. CD32-expressing CD4 T cells are phenotypically diverse and can contain proviral HIV DNA. Front Immunol. (2018) 9:928. 10.3389/fimmu.2018.00928 29780387 PMC5946760

[17] Engelhardt W Matzke J Schmidt RE. Activation-dependent expression of low affinity IgG receptors Fc gamma RII(CD32) and Fc gamma RIII(CD16) in subpopulations of human T lymphocytes. Immunobiology (1995) 192:297–320. 7649565 10.1016/s0171-2985(11)80172-5

[18] Sandilands GP MacPherson SA Burnett ER Russell AJ Downie I MacSween RN. Differential expression of CD32 isoforms following alloactivation of human T cells. Immunology (1997) 91:204–11. 9227318 10.1046/j.1365-2567.1997.00241.x PMC1363848

[19] Sandilands GP McLaren AP Howie D MacSween RN. Occult expression of CD32 (Fc gamma RII) in normal human peripheral blood mononuclear cells. Immunology (1995) 86:525–32. 8567016 PMC1384050

[20] Sandilands GP Burnett ER MacPherson SA Downie I More IA MacSween RN. Demonstration of cytoplasmic CD32 (Fc gamma RII) within human lymphocytes following microwave treatment. Immunology (1997) 90:427–34. 9155651 10.1111/j.1365-2567.1997.00427.x PMC1456611

[21] Abdel-Mohsen M Kuri-Cervantes L Grau-Exposito J Spivak AM Nell RA Tomescu C CD32 is expressed on cells with transcriptionally active HIV but does not enrich for HIV DNA in resting T cells. Sci Transl Med. (2018) 10:eaar6759. 10.1126/scitranslmed.aar6759 PMC6282755 29669853

[22] Theofilopoulos AN Dixon FJ. Immune complexes in human diseases: a review. Am J Pathol. (1980) 100:529–94. 6157327 PMC1903541

[23] Celis E Chang TW. HBsAg-serum protein complexes stimulate immune T lymphocytes more efficiently than do pure HBsAg. Hepatology (1984) 4:1116–23. 6238896 10.1002/hep.1840040604

[24] Xu DZ Huang KL Zhao K Xu LF Shi N Yuan ZH. Vaccination with recombinant HBsAg-HBIG complex in healthy adults. Vaccine (2005) 23:2658–64. 10.1016/j.vaccine.2004.10.040 15780449

[25] Heyman B. Antibodies as natural adjuvants. Curr Top Microbiol Immunol. (2014) 382:201–19. 10.1007/978-3-319-07911-0_9 25116101

[26] Pelegrin M Naranjo-Gomez M Piechaczyk M. Antiviral monoclonal antibodies: can they be more than simple neutralizing agents? Trends Microbiol. (2015) 23:653–65. 10.1016/j.tim.2015.07.005 26433697 PMC7127033

[27] Lambour J Naranjo-Gomez M Piechaczyk M Pelegrin M. Converting monoclonal antibody-based immunotherapies from passive to active: bringing immune complexes into play. Emerg Microbes Infect. (2016) 5:e92. 10.1038/emi.2016.97 27530750 PMC5034104

[28] Wen YM Mu L Shi Y. Immunoregulatory functions of immune complexes in vaccine and therapy. EMBO Mol Med. (2016) 8:1120–33. 10.15252/emmm.201606593 27572622 PMC5048363

[29] Bournazos S Wang TT Dahan R Maamary J Ravetch JV. Signaling by antibodies: recent progress. Annu Rev Immunol. (2017) 35:285–311. 10.1146/annurev-immunol-051116-052433 28446061 PMC5613280

[30] Maamary J Wang TT Tan GS Palese P Ravetch JV. Increasing the breadth and potency of response to the seasonal influenza virus vaccine by immune complex immunization. Proc Natl Acad Sci USA. (2017) 114:10172–7. 10.1073/pnas.1707950114 28874545 PMC5617290

[31] van Mirre E Breunis WB Geissler J Hack CE de Boer M Roos D. Neutrophil responsiveness to IgG, as determined by fixed ratios of mRNA levels for activating and inhibitory FcgammaRII (CD32), is stable over time and unaffected by cytokines. Blood (2006) 108:584–90. 10.1182/blood-2005-12-4997 16551965

[32] Banki Z Kacani L Mullauer B Wilflingseder D Obermoser G Niederegger H. Cross-linking of CD32 induces maturation of human monocyte-derived dendritic cells via NF-kappa B signaling pathway. J Immunol. (2003) 170:3963–70. 10.4049/jimmunol.170.8.3963 12682223

[33] Brooks DG Qiu WQ Luster AD Ravetch JV. Structure and expression of human IgG FcRII(CD32). Functional heterogeneity is encoded by the alternatively spliced products of multiple genes. J Exp Med. (1989) 170:1369–85. 2529342 10.1084/jem.170.4.1369 PMC2189488

[34] de Andres B Mueller AL Verbeek S Sandor M Lynch RG. A regulatory role for Fcgamma receptors CD16 and CD32 in the development of murine B cells. Blood (1998) 92:2823–9. 9763567

[35] Boruchov AM Heller G Veri MC Bonvini E Ravetch JV Young JW. Activating and inhibitory IgG Fc receptors on human DCs mediate opposing functions. J Clin Invest. (2005) 115:2914–23. 10.1172/JCI24772 16167082 PMC1201664

[36] Avery L Filderman J Szymczak-Workman AL Kane LP. Tim-3 co-stimulation promotes short-lived effector T cells, restricts memory precursors, and is dispensable for T cell exhaustion. Proc Natl Acad Sci USA. (2018) 115:2455–60. 10.1073/pnas.1712107115 29463725 PMC5877951

[37] Roberts GM Davies EV Pettit EJ Hallett MB. The timing and magnitude of Ca2+ signaling by CD32 depends on its redistribution on the cell surface. Exp Cell Res. (1997) 230:303–9. 10.1006/excr.1996.3416 9024789

[38] Arman M Krauel K. Human platelet IgG Fc receptor FcgammaRIIA in immunity and thrombosis. J Thromb Haemost. (2015) 13:893–908. 10.1111/jth.12905 25900780

[39] Ghazizadeh S Bolen JB Fleit HB. Tyrosine phosphorylation and association of Syk with Fc gamma RII in monocytic THP-1 cells. Biochem J. (1995) 305(Pt 2):669–74. 7530449 10.1042/bj3050669 PMC1136413

[40] Burchill MA Yang J Vogtenhuber C Blazar BR Farrar MA. IL-2 receptor beta-dependent STAT5 activation is required for the development of Foxp3+ regulatory T cells. J Immunol. (2007) 178:280–90. 10.4049/jimmunol.178.1.280 17182565

[41] O'Shea JJ Paul WE. Mechanisms underlying lineage commitment and plasticity of helper CD4+ T cells. Science (2010) 327:1098–02. 10.1126/science.1178334 20185720 PMC2997673

[42] Takai T. Roles of Fc receptors in autoimmunity. Nat Rev Immunol. (2002) 2:580–92. 10.1038/nri856 12154377

[43] Muta T Kurosaki T Misulovin Z Sanchez M Nussenzweig MC Ravetch JV. A 13-amino-acid motif in the cytoplasmic domain of Fc gamma RIIB modulates B-cell receptor signalling. Nature (1994) 368:70–3. 10.1038/368070a0 8107887

[44] Xiang Z Cutler AJ Brownlie RJ Fairfax K Lawlor KE Severinson E. FcgammaRIIb controls bone marrow plasma cell persistence and apoptosis. Nat Immunol. (2007) 8:419–29. 10.1038/ni1440 17322888

[45] Kliks S. Antibody-enhanced infection of monocytes as the pathogenetic mechanism for severe dengue illness. AIDS Res Hum Retroviruses (1990) 6:993–8. 10.1089/aid.1990.6.993 2223245

[46] Hessell AJ Hangartner L Hunter M Havenith CE Beurskens FJ Bakker JM Fc receptor but not complement binding is important in antibody protection against HIV. Nature (2007) 449:101–4. 10.1038/nature06106 17805298

[47] Monsalvo AC Batalle JP Lopez MF Krause JC Klemenc J Hernandez JZ. Severe pandemic 2009 H1N1 influenza disease due to pathogenic immune complexes. Nat Med. (2011) 17:195–9. 10.1038/nm.2262 21131958 PMC3034774

[48] Ohyama K Ueki Y Kawakami A Kishikawa N Tamai M Osaki M. Immune complexome analysis of serum and its application in screening for immune complex antigens in rheumatoid arthritis. Clin Chem. (2011) 57:905–9. 10.1373/clinchem.2010.157776 21482748

[49] Moore TL. Immune complexes in juvenile idiopathic arthritis. Front Immunol. (2016) 7:177. 10.3389/fimmu.2016.00177 27242784 PMC4873492

[50] Mancini I Ferrari B Valsecchi C Pontiggia S Fornili M Biganzoli E. ADAMTS13-specific circulating immune complexes as potential predictors of relapse in patients with acquired thrombotic thrombocytopenic purpura. Eur J Intern Med. (2017) 39:79–83. 10.1016/j.ejim.2016.11.003 27887777

[51] Olaru F Dobel T Lonsdorf AS Oehrl S Maas M Enk AH. Intracapillary immune complexes recruit and activate slan-expressing CD16+ monocytes in human lupus nephritis. JCI Insight (2018) 3:96492. 10.1172/jci.insight.96492 29875315 PMC6124405

[52] Gamberale R Geffner JR Giordano M. Immune complexes and apoptosis in B-cell chronic lymphocytic leukemia. Leuk Lymphoma (2002) 43:251–5. 10.1080/10428190290006008 11999554

[53] Dhodapkar KM Kaufman JL Ehlers M Banerjee DK Bonvini E Koenig S. Selective blockade of inhibitory Fcgamma receptor enables human dendritic cell maturation with IL-12p70 production and immunity to antibody-coated tumor cells. Proc Natl Acad Sci USA. (2005) 102:2910–5. 10.1073/pnas.0500014102 15703291 PMC549508

[54] Cramer DW O'Rourke DJ Vitonis AF Matulonis UA Dijohnson DA Sluss PM. CA125 immune complexes in ovarian cancer patients with low CA125 concentrations. Clin Chem. (2010) 56:1889–92. 10.1373/clinchem.2010.153122 20943848 PMC3079311

[55] Lanier LL Kipps TJ Phillips JH. Functional properties of a unique subset of cytotoxic CD3+ T lymphocytes that express Fc receptors for IgG (CD16/Leu-11 antigen). J Exp Med. (1985) 162:2089–106. 2415663 10.1084/jem.162.6.2089 PMC2187997

[56] Chauhan AK Chen C Moore TL DiPaolo RJ. Induced expression of FcgammaRIIIa (CD16a) on CD4+ T cells triggers generation of IFN-gammahigh subset. J Biol Chem. (2015) 290:5127–40. 10.1074/jbc.M114.599266 25556651 PMC4335247

[57] Chauhan AK Moore TL Bi Y Chen C. FcgammaRIIIa-Syk Co-signal modulates CD4+ T-cell response and up-regulates Toll-like Receptor (TLR) expression. J Biol Chem. (2016) 291:1368–86. 10.1074/jbc.M115.684795 26582197 PMC4714221

[58] Chauhan AK A contribution of FcγRIIIa cosignaling in T_FH_ subset development in Systemic Lupus Erythematosus. bioRxiv (2018) 256198:1–31. 10.1101/256198

